# The Design and Fabrication of a MEMS Electronic Calibration Chip

**DOI:** 10.3390/mi13122139

**Published:** 2022-12-03

**Authors:** Qiannan Wu, Yu Chen, Qianlong Cao, Jingchao Zhao, Shanshan Wang, Junqiang Wang, Mengwei Li

**Affiliations:** 1 School of Semiconductors and Physics, North University of China, Taiyuan 030051, China; 2 Center for Microsystem Intergration, North University of China, Taiyuan 030051, China; 3 Academy for Advanced Interdisciplinary Research, North University of China, Taiyuan 030051, China; 4 School of Instrument and Intelligent Future Technology, North University of China, Taiyuan 030051, China; 5 School of Instrument and Electronics, North University of China, Taiyuan 030051, China; 6 Key Laboratory of Dynamic Measurement Technology, North University of China, Taiyuan 030051, China

**Keywords:** calibration, RF MEMS, integration, TaN Resistors, switches

## Abstract

During the test of microelectromechanical system (MEMS) devices, calibration of test cables, loads and test instruments is an indispensable step. Calibration kits with high accuracy, great operability and small loss can reduce the systematic errors in the test process to the greatest extent and improve the measurement accuracy. Aiming at the issues of the conventional discrete calibration piece unit, which presents cumbersome calibration steps and large system loss, an integrated electronic calibration chip based on frequency microelectromechanical system (RF MEMS) switches is designed and fabricated. The short-open-load-through (SOLT) calibration states can be completed on a single chip, step by step, by adjusting the on–off state of the RF MEMS switches. The simulation results show that the operating frequency of the electronic calibration piece covers the range of DC~26.5 GHz, the insertion loss in through (thru) state is less than 0.2 dB, the return loss is less than 1.0 dB in short-circuit and open-circuit states, the return loss under load-circuit state is less than 20 dB and its size is only 2.748 mm × 2.2 mm × 0.5 mm. This novel calibration chip design has certain esteem for advancing calibration exactness and effectiveness.

## 1. Introduction

With the development of technologies such as microwave communication and the Internet of Things, there are more and more demands for high-performance RF microwave devices and services, such as broadband and low power consumption [1]. The market application value of RF MEMS devices has been greatly improved. However, during the test of MEMS device parameters, microwave test cables and loads had problems such as large loss, and instability, which directly lead to lower test accuracy. Therefore, it is necessary to calibrate test cables, loads and instruments before testing to minimize systematic errors during testing and improve measurement accuracy [2].

With the development of RF devices towards high frequency and integration [3], the test of devices’ S-parameters, power, gain, noise and other parameters is more reliant on the calibration of the on-chip S-parameters. Domestic and foreign scientific research institutions have carried out a lot of research on device testing. With the development of device on-chip testing [4], in 2014, the Fourth Institute of Electronics developed a ceramic-based DC~40 GHz calibration chip, which can meet the chip calibration requirements based on the SOLT calibration principle [5]. Although the frequency band of the calibration chip is wide, the calibration unit is discrete, and the testing procedure is tedious. In 2017, the Institute of Electronic Standardization designed a discrete calibration sample based on GaAs substrate, which can meet the on-chip testing requirements of GaAs devices [6]. In 2021, North Central University designed a RF MEMS calibration component based on the SOLT calibration principle, which meets the calibration requirements for chip-on-chip testing in the DC~20 GHz range [7]. The quasi chip integrates the RF MEMS switch with low insertion loss and high isolation performance, which has a certain significance for improving calibration accuracy and reducing test complexity. However, the precision chip is slightly lower in frequency and larger in size.

Aiming at the problems of discrete units and low work efficiency in traditional calibration samples [8,9,10], this paper designs an integrated calibration sample based on RF MEMS switches, which can be used for the calibration of on-chip test systems of RF and microwave devices to ensure the accuracy of device performance calibration and improve calibration [11,12,13]. Work efficiency is of great significance to the automation of the on-chip calibration system.

## 2. Structural Design of Calibration Sheet

**Figure 1:**
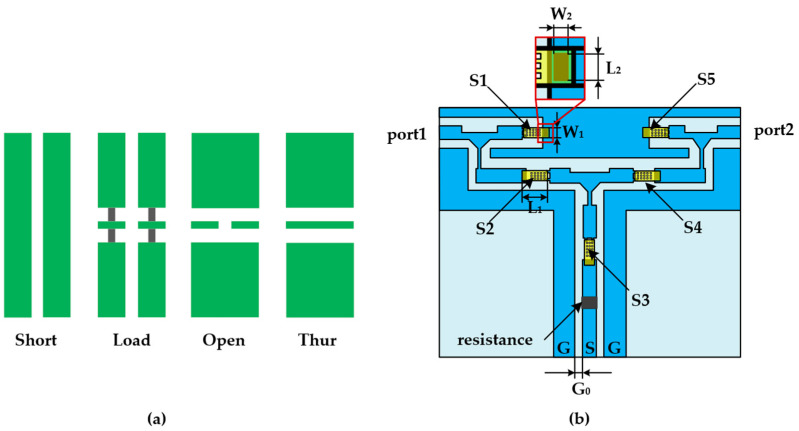
Calibration element structure diagram: (**a**) Discrete calibration unit; (**b**) structure of integrated electronic calibration chip based on RF MEMS switches.

The calibration chip is designed based on the SOLT calibration principle, and it employs RF MEMS switches to realize the integration of four calibration states: through, load, open circuit and short circuit. The currently used calibration sample is shown in Figure 1a. Each calibration unit adopts a discrete design. During calibration, it is necessary to move the probe several times for measurement.

The device structure of the designed integrated electronic calibration chip is shown in Figure 1b in this paper. The chip presents a symmetrical structure, and S1–S5 represent RF MEMS switches. Structural design parameters are shown in Table 1. The device uses a coplanar waveguide as a signal transmission line and a tantalum nitride thin-film resistor as a load resistor [14,15]. By applying a DC control voltage, the state of the RF MEMS switch is controlled to achieve switching between different calibration states [16]. When calibrating port 1, the signal is grounded by controlling the switch S1 to realize the short-circuit calibration state. An open calibration state is achieved when all switches are open. When switches S2 and S3 are controlled so that port 1 is connected to the load resistance, the load calibration state is realized. When switches S2 and S4 are controlled, a through calibration state between port 1 and port 2 is achieved. Similarly, the calibration process of port 2 is the same as above. The corresponding switch mode of the calibration state is described in Table 2.

**Figure 2:**
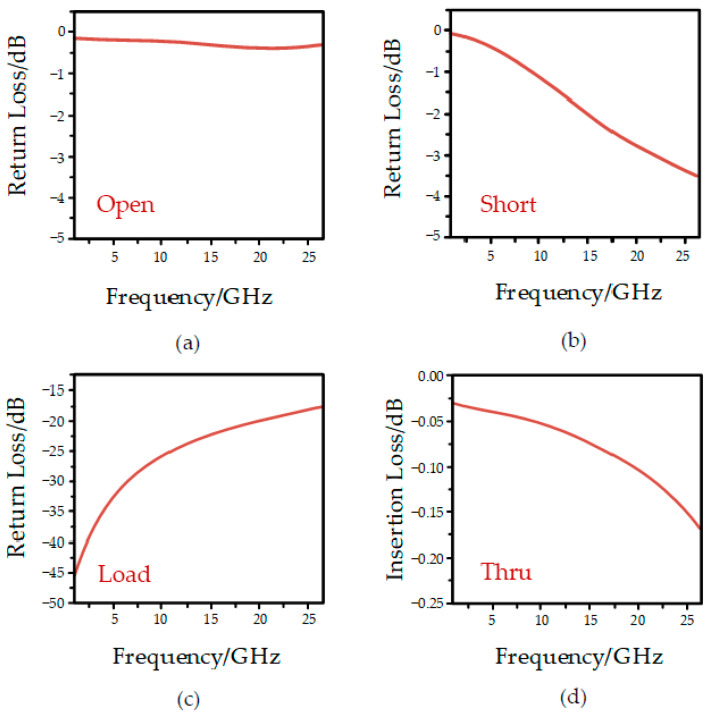
Simulation results in different states: (**a**) Open circuit; (**b**) short circuit; (**c**) load circuit; (**d**) Thru circuit.

The device uses ANSYS HFSS software for numerical simulation analysis. When port 1 is calibrated in the range of DC~26.5 GHz, the simulation results of different states are shown in Figure 2 below. As can be seen in the Figure, when the device is in open circuit and short circuit, its return loss is less than 3.5 dB, and the microwave signal is in a state of total reflection. When the device is in the load calibration state, its return loss is less than 20 dB, and the signal is basically absorbed by the load. In the direct-through calibration phase, the signal is transmitted at low loss through the calibration piece, realizing the direct connection of the two test ports, and the insertion loss S21 is less than 0.20 dB and close to 0 dB.

## 3. Device Fabrication Process Flow

**Figure 3:**
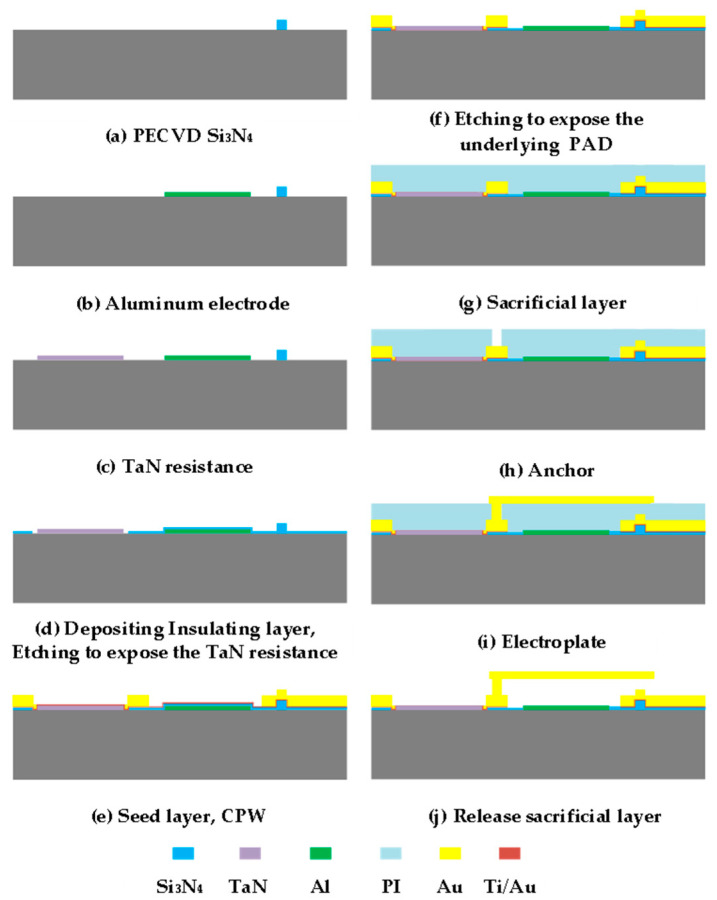
Device process flow.

**Figure 4:**
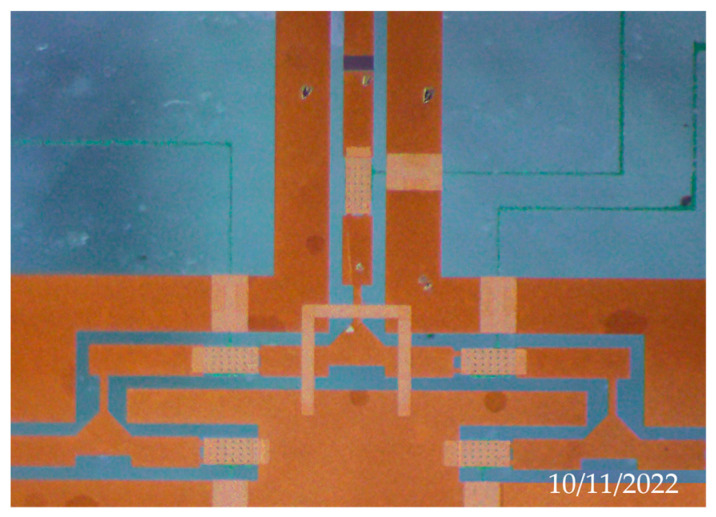
Optical photo of device.

**Figure 5:**
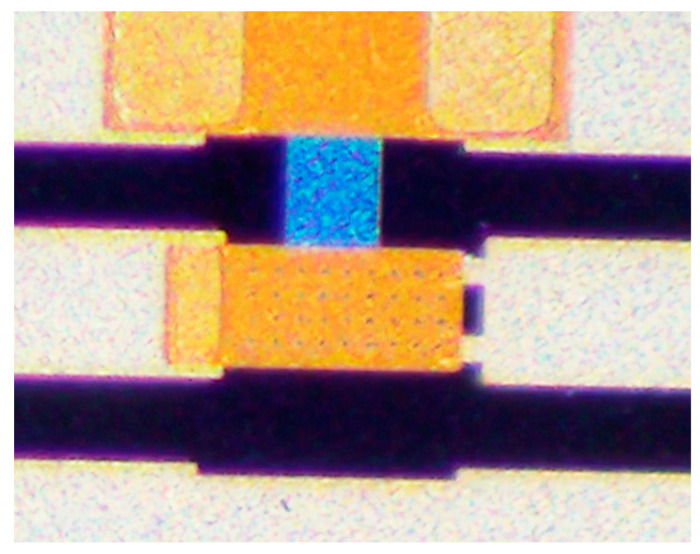
Details of RF MEMS switch in calibrator.

Figure 3 is a process flow diagram of an electronic calibration chip based on a RF MEMS switch. The device uses borophosphosilicate glass (dielectric constant of 4.9) as the substrate material. A 500 nm thin film of silicon nitride (Si_3_N_4_) was grown at 350 °C via the PECVD process and etched to form switch contacts, as shown in Figure 3a. Next, 500 nm aluminum (Al) was grown via the magnetron sputtering process and etched in phosphoric acid solution (H_3_PO_4_) at 50 °C to form the switch-driving electrodes, as described in Figure 3b. Under the condition of RF power of 300 W and airflow of 200 sccm, tantalum nitride film was grown via magnetron sputtering for 10 min as the load resistor in Figure 3c. A silicon nitride film of 300 nm was grown as an isolation layer to avoid electrical breakdown easily occurring during device operation, and exposing the TaN resistance by etching, shown in Figure 3d. Additionally, 50 nm/150 nm Ti/Au was grown as a seed layer, and 2μm gold was prepared via a micro-plating process as a coplanar waveguide for RF signal transmission in Figure 3e. A Ti/Au seed layer was removed via wet etching. The silicon nitride isolation layer was etched by photolithography and dry etching to expose the underlying PAD in Figure 3f. From Figure 3g, spin-coated polyimide was used as the sacrificial layer of the device, and precured at 80 °C for 5 h. After the anchors in the sacrificial layer were obtained by a mask and wet etching, the sacrificial layer was cured at 300 °C in Figure 3h. Then, 100 nm gold was sputtered as the seed layer, and the top electrode of the switch was fabricated by a micro-electroplating process at 60 °C, as shown in Figure 3i. Under the condition of radio frequency power of 400 W, the sacrificial layer was etched by oxygen plasma to release the sacrificial layer, shown in Figure 3j. The surface structure of the device prepared by the surface micromachining process is shown in Figure 4, and the detailed structure of the RF MEMS switch in the device is shown in Figure 5.

## 4. Device Performance Test

**Figure 6:**
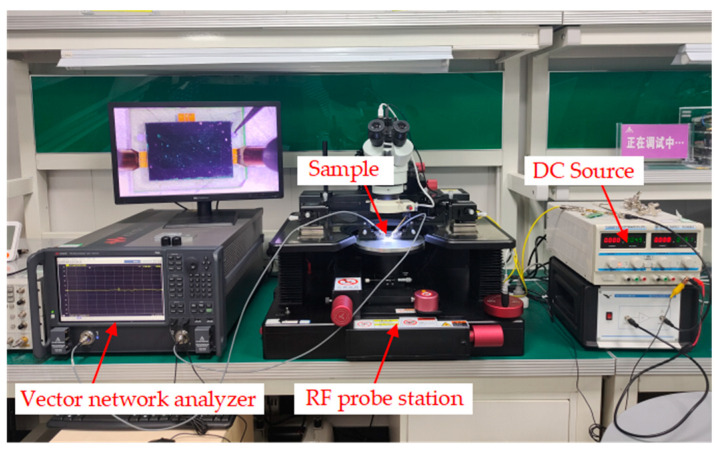
Microwave performance test of the MEMS electronic calibration chip.

**Figure 7:**
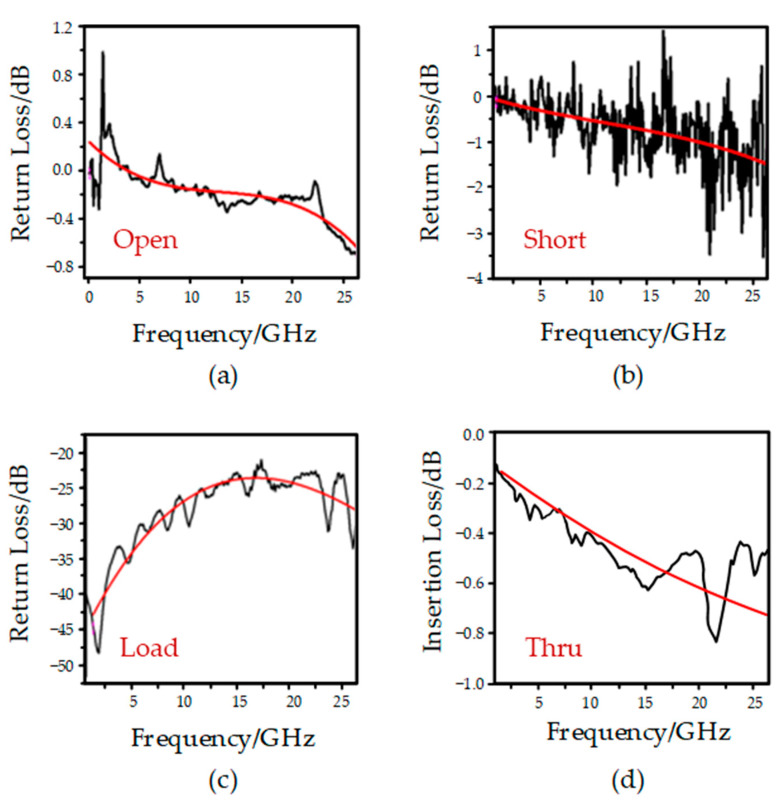
Test results for each status: (**a**) Open circuit; (**b**) short circuit; (**c**) load circuit; (**d**) Thur circuit.

The microwave test system is shown in Figure 6. As can be seen from the figure, the electronic calibration chip is connected to the vector network analyzer through the RF probe and the RF transmission line. In addition, the signal from the DC voltage source passes through the voltage amplifier to the actuation electrode of the switch on the electronic calibration chip. When the applied voltage reaches the driving voltage of the switch, the upper electrode moves vertically downward under the action of electrostatic force, the switch is closed and the signal is turned on. Thus, the calibration state is switched by controlling the RF MEMS switch on or off for each channel. The performance of the device in different states is characterized by the vector network analyzer. The test results are shown in Figure 7. In the open circuit state, the return loss of port 1 is less than 0.7 dB; in the short-circuit state, the echo state of port 1 is less than 3.5 dB. In the load state, the return loss of port 1 is less than 25 dB, which is small compared with the simulation results and meets the design requirements. In the pass-through state, the insertion loss between the ports is less than 1 dB, which is slightly greater than the design result, which is the result of the loss of the test environment and the probe front end. The port isolation is greater than 30 dB, which meets the design results. The reasons for the positive return loss may include two aspects. On the one hand, the surrounding electromagnetic environment is not shielded. On the other hand, it is caused by the poor contact between the probe and the calibration chip, as well as the connection line and the port of the vector network analyzer.

Table 3 provides the comparison between the simulation results and the test results of the MEMS electronic calibration chip designed in this paper. It can be seen that the test results are basically consistent with the simulation results. The deviation is caused by uncontrollable factors present during processing. Table 4 provides a comparison between the results for currently used calibration chips and the electronic calibration chip designed in this study. As can be seen from Table 3, the proposed electronic calibration component achieves the conversion of different calibration states by controlling the on–off state of RF MEMS switch, thus improving the efficiency of calibration. Compared with the previous calibration devices, the proposed electronic calibration component has the advantages of less loss, small size and high integration.

## 5. Conclusions

In this paper, the electronic calibration chips in four calibration states are proposed. They are composed of the three cascaded MEMS SPDT switches and one load resistance. The MEMS calibration chip can not only meet the calibration accuracy and efficiency of device on-chip testing, but also provide reduced size, loss and calibration cost. It has certain application value in microwave instruments, on-chip testing and other fields.

## Figures and Tables

**Table 1:** Device structure parameters.

S.no	Design Parameter	Values/μm
1	CPW (G-S-G)	75-120-75
2	Bridge length (W_1_)	250
3	Bridge width (L_1_)	100
4	Signal line gap (G_0_)	3
5	Anchor length (L_2_)	80
6	Anchor width (W_2_)	20

**Table 2:** Working principle table.

State	S1	S2	S3	S4
thru	0	1	0	1
open	0	0	0	0
short	1	0	0	0
load	0	1	1	0

Note: 1 means the switch is closed; 0 means the switch is open.

**Table 3:** Comparison of simulation results and test results.

State of Calibration	Simulation Result	Test Result
Open	S11 < 1.0 dB	S11 < 0.7 dB
Short	S11 < 3.5 dB	S11 < 3.5 dB
Load	S11 < 20 dB	S11 < 25 dB
Thru	S21 < 0.20 dB	S21 < 1.0 dB

**Table 4:** Comparison with previous work.

Years	2014 [5]	2017 [6]	2021 [7]	This Paper
Frequency (GHz)	40	50	20	26.5
Size (mm)	--	--	6.00 × 2.80 × 0.80	2.748 × 2.200 × 0.50
Material	Ceramic	GaAs	glass	glass
Principle	SOLT	SOLT	SOLT	SOLT
Type	Discrete	Discrete	Integrated	Integrated

## Data Availability

The data that support the findings of this study are available from the corresponding author upon reasonable request.
